# Shipboard design and fabrication of custom 3D-printed soft robotic manipulators for the investigation of delicate deep-sea organisms

**DOI:** 10.1371/journal.pone.0200386

**Published:** 2018-08-01

**Authors:** Daniel M. Vogt, Kaitlyn P. Becker, Brennan T. Phillips, Moritz A. Graule, Randi D. Rotjan, Timothy M. Shank, Erik E. Cordes, Robert J. Wood, David F. Gruber

**Affiliations:** 1 Wyss Institute for Biologically Inspired Engineering, Harvard University, Cambridge, MA, United States of America; 2 Harvard John A. Paulson School of Engineering and Applied Sciences, Harvard University, Cambridge, MA, United States of America; 3 Department of Ocean Engineering, University of Rhode Island, Narragansett, RI, United States of America; 4 Department of Biology, Boston University, Boston, MA, United States of America; 5 Woods Hole Oceanographic Institution, Woods Hole, MA, United States of America; 6 Department of Biology, Temple University, Philadelphia, PA, United States of America; 7 Department of Natural Sciences, Baruch College and The Graduate Center PhD Program in Biology, City University of New York, New York, NY, United States of America; 8 Radcliffe Institute for Advanced Study, Harvard University, Cambridge, MA, United States of America; Universita del Salento, ITALY

## Abstract

Soft robotics is an emerging technology that has shown considerable promise in deep-sea marine biological applications. It is particularly useful in facilitating delicate interactions with fragile marine organisms. This study describes the shipboard design, 3D printing and integration of custom soft robotic manipulators for investigating and interacting with deep-sea organisms. Soft robotics manipulators were tested down to 2224m via a Remotely-Operated Vehicle (ROV) in the Phoenix Islands Protected Area (PIPA) and facilitated the study of a diverse suite of soft-bodied and fragile marine life. Instantaneous feedback from the ROV pilots and biologists allowed for rapid re-design, such as adding “fingernails”, and re-fabrication of soft manipulators at sea. These were then used to successfully grasp fragile deep-sea animals, such as goniasterids and holothurians, which have historically been difficult to collect undamaged via rigid mechanical arms and suction samplers. As scientific expeditions to remote parts of the world are costly and lengthy to plan, on-the-fly soft robot actuator printing offers a real-time solution to better understand and interact with delicate deep-sea environments, soft-bodied, brittle, and otherwise fragile organisms. This also offers a less invasive means of interacting with slow-growing deep marine organisms, some of which can be up to 18,000 years old.

## Introduction

Marine biologists studying deep sea environments are confronted with technological difficulties while gaining access to, interacting with, and collecting marine life. Beyond the limits of technical scientific scuba diving (150m), it is necessary for submersible vehicles such as ROVs, manned submersibles, or Autonomous Underwater Vehicles (AUVs) to access, observe and interact with deep-marine environments. Using these platforms, marine biologists have primarily utilized suction samplers, rigid canisters, and industrial robotic manipulator arms, which are generally made of inflexible metals. These devices have been designed primarily for the offshore energy industry or military applications and are often not suitable for interacting with soft-bodied and highly fragile organisms. For several decades, marine biologists have been trying to grasp megafaunal organisms without damaging them using a traditional hard-bodied robot hand or claw. It is important to minimize damage to deep-sea samples, as many are vulnerable organisms with slow growth rates, and long life spans. For instance, a deep-sea black coral was recently aged at 4,625 years old [1], while a sponge was aged at ∼18,000 years old [2].

Soft manipulators have previously shown their utility for underwater biological sampling [3][4][5]. Soft manipulators are constructed out of compliant materials instead of rigid elements [6]. The use of soft materials offers the advantage of simplifying the manipulator’s control, e.g., by leveraging mechanical compliance such that knowledge of the exact position and dimension of the desired object is not required. The soft manipulator can automatically conform to its shape with minimal applied forces. Additionally, soft-bodied manipulators have the advantage of not damaging delicate specimens with sharp edges or inflexible grasps. It has recently been shown that soft robots could be successfully used in deep sea environments down to 800m [7]. And, subsequently, a modular soft robotic wrist [8] and an entire soft robotic arm have been developed [9].

3D printing offers numerous kinds of fabrication processes, such as Stereolithography (SLA), Selective Laser Sintering (SLS), and many more as described in [10]. 3D printing has been widely democratized and allows engineers, citizen scientists, and hobbyists to 3D print objects at home. Along with plastics, 3D printing can also be used with composites [11], wax, and edible [12] materials. Yet, when 3D printing soft materials [13][14], additional challenges of potential self-collapse must be overcome to prevent the 3D printed structure from deforming under its own weight [15]. Researchers have been able to overcome this challenge and 3D print soft robots using polyjet [16], stereolithography [17], or Fused Deposition Modeling [18] (FDM) technologies.

**Fig 1:**
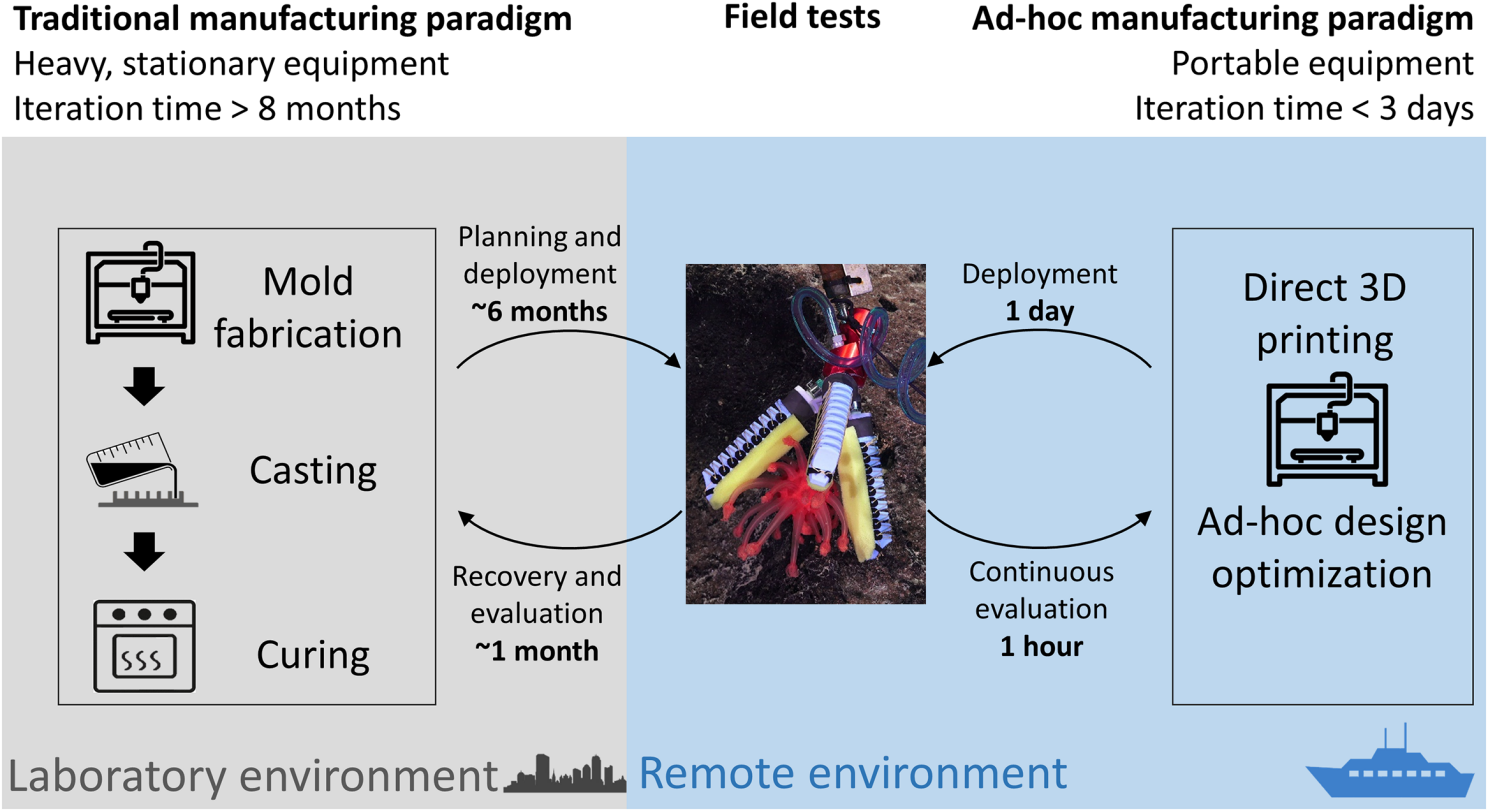
Comparison between traditional and in-field manufacturing of soft manipulators. The ability to iterate the design and fabricate actuators in-field is key to enable adaptations to specific and unanticipated challenges found in unstructured, remote environments (indicated by the blue background).

When conducting ocean exploration on research vessels in highly remote areas, lab-manufactured tools can be limiting, as it is difficult to predict engineering scenarios in advance. Additionally, while many such scenarios can be anticipated, there is limited space on board to accommodate the necessary tools. The ability to generate in-field adaptive strategies is one real-time solution to meet the needs and conditions encountered while using minimal space on board for materials and fabrication tools. A comparison of traditional and ad-hoc manufacturing of a soft device is shown in Fig 1. 3D printing has already shown its potential to be used in unconventional places such as providing medical tools in remote mountain hospitals [19] or even in space [20].

During this deep-sea expedition to the Phoenix Islands Protected Area from October 5^th^ to November 2^nd^ 2017, traditionally molded soft manipulators were first used for sampling and later modified over consecutive dives with feedback from biologists and ROV pilots. This real-time end-user feedback catalyzed the on-board design and fabrication of a new soft manipulator that was tested during the subsequent dives to sample species such as goniasterids and holothurians down to 2224m.

## Materials and methods

### Expedition and deep-sea sampling

**Fig 2:**
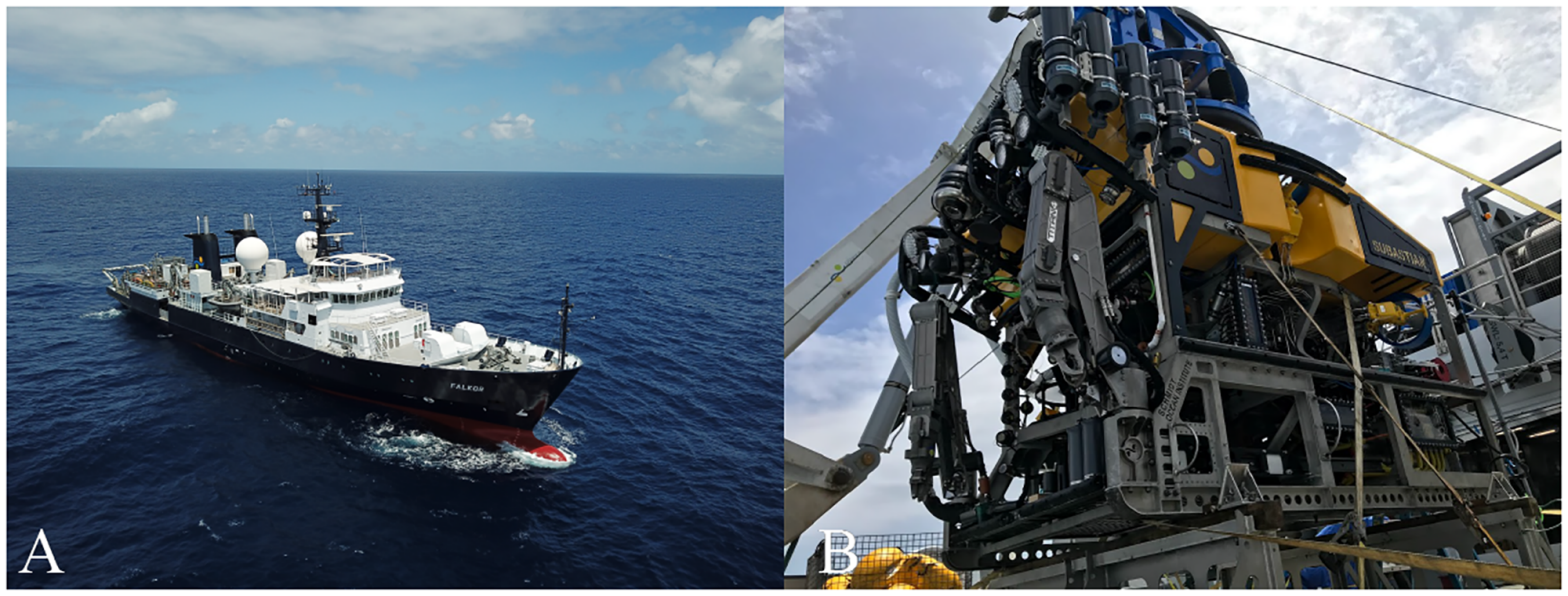
Research vessel and the remotely operated underwater vehicle. A: R/V *Falkor*. B: ROV *SuBastian*.

**Fig 3:**
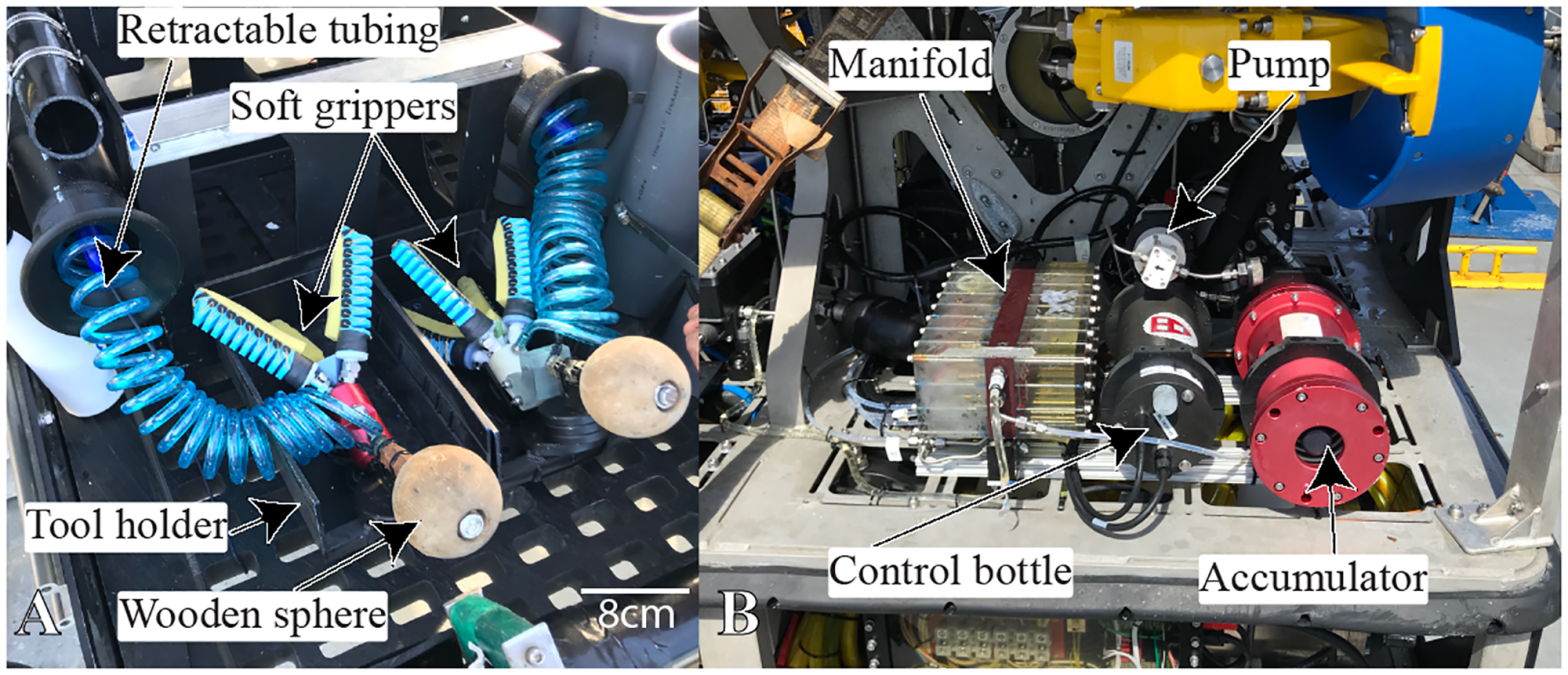
Soft manipulator setup on the ROV. A: The soft manipulators are installed on the retractable tray of the ROV. B: The manifold, pump, control bottle, and accumulator are installed on the port rear side. These were developed as part of a previous study [9].

The Phoenix Islands (Republic of Kiribati) are located in the central Pacific and include a total of eight islands, two shallow submerged seamounts, and a diverse array of deep seamounts including the Tokelau Chain. It is the largest and deepest UNESCO World Heritage site on earth [21] and offers a unique environment with high regional biodiversity and little or no human activity. The R/V *Falkor* and the ROV *SuBastian* (Schmidt Ocean Institute, Figs 2A and 3B), visited the PIPA seamounts to investigate deepwater corals, coral invertebrate epifauna, and sediments. Most sites targeted during this expedition were completely unexplored.

**Fig 4:**
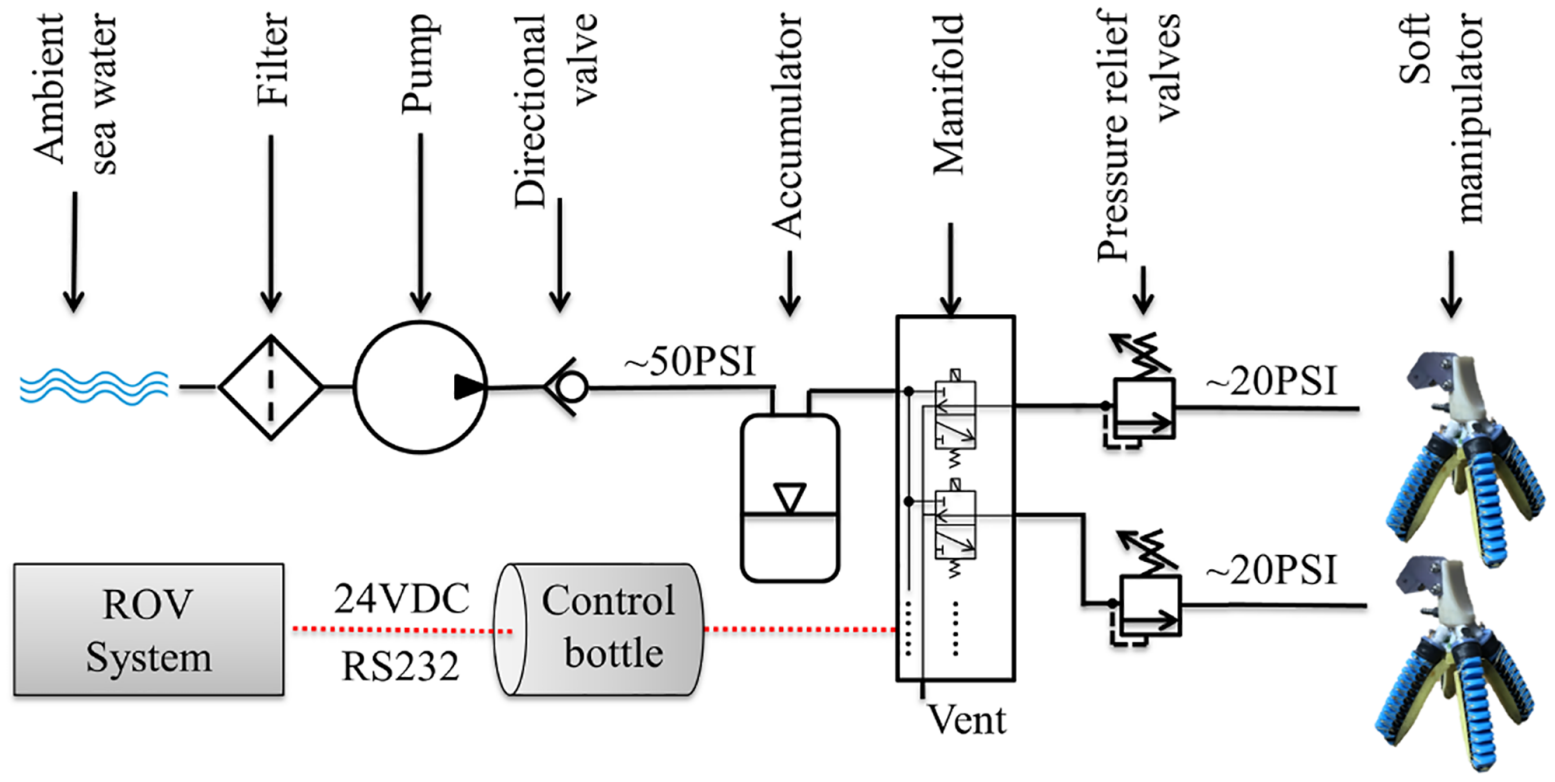
Full schematic of the soft manipulator setup. Hydraulic (solid black line) and electrical connections (dashed red lines) are represented. This setup is similar to the one used in [9].

To collect samples, the ROV *SuBastian* (rated to 4500m) was equipped with several standard tools including two heavy-duty manipulator arms and a hydraulic suction sampler. Each Schilling Robotics TITAN 4 Manipulator arm on SuBastian ROV was equipped with a distal manipulator with jaws which could open to 186.74mm and exert a maximum nominal grip force of 4092 Newtons. During this expedition, we initially employed soft manipulators developed at the Harvard Microrobotics Laboratory in Cambridge, Massachusetts. Lessons learned and real-time feedback from the ROV pilots and scientists then guided the subsequent design and fabrication of different 3D-printed manipulators, which were used during the following dives. The soft manipulator setup [9] brought on this expedition was organized into two parts. The soft grippers (Fig 3A) were installed in holsters on the ROV’s forward hydraulic tray. They were in reach of the port robotic four-fingered manipulator, which was able to lock onto a wooden ball (acting as a handle) connected to the soft manipulator gripper. Retractable tubing provided low-pressure hydraulics to the soft manipulators. The remaining equipment required to use the soft manipulators (pump, manifold, etc.) was positioned on the rear side of the ROV (Fig 3B). The pump filled the accumulator with ambient seawater, which powered the manifold. A control bottle contained the electronic circuitry to communicate with both the surface controls (serial communication, RS-232), and proportionally activate the solenoid valves in the manifold. An adjustable pressure relief valve located between the manifold and the soft gripper ensured that the actuators were not over-pressurized. The full diagram of the electrical and hydraulic connections is shown in Fig 4.

### Laboratory-fabricated soft manipulators

**Fig 5:**
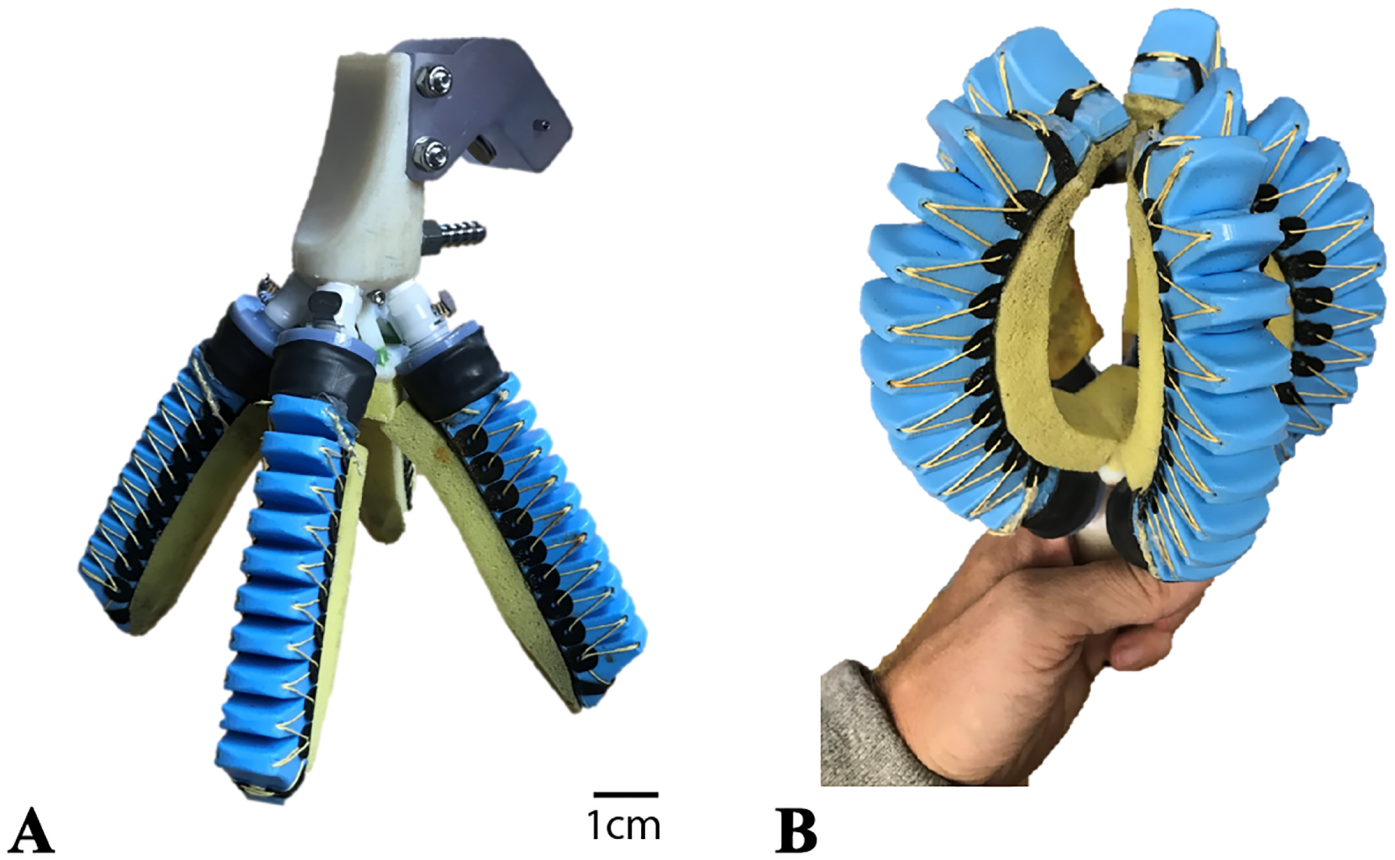
A traditionally laboratory-fabricated soft manipulator. A: Open/deflated configuration. B: Closed/inflated configuration.

Fluidic soft actuators operate on a pressure differential (pneumatic or hydraulic) between the interior chambers and the surrounding pressure (Fig 5). Soft actuators are traditionally laboratory-fabricated and require several steps of molding to accommodate curing times of the constituent elastomeric materials [22][23]. Although this method has proven effective, the long curing times and equipment required (e.g., vacuum chamber, oven, and mixing equipment) makes the method inefficient for in-field fabrication and rapid prototyping. These actuators are based on the bellows-style actuators (with foam pads) described in [7]. When lifting up an object, one can expect a maximum pulling force of 16.6 Newtons before the actuator drops the object. Due to the foam layers, typical pressures applied to the object are usually limited to 6kPa, equivalent to the approximate pressure required to activate a keyboard button. For a typical operating pressure of 140kPa, the actuator can exert a blocked force of 0.96N and can grasp objects up to 140mm in diameter (S1 Fig).

### Ad-hoc 3D printed grippers

**Fig 6:**
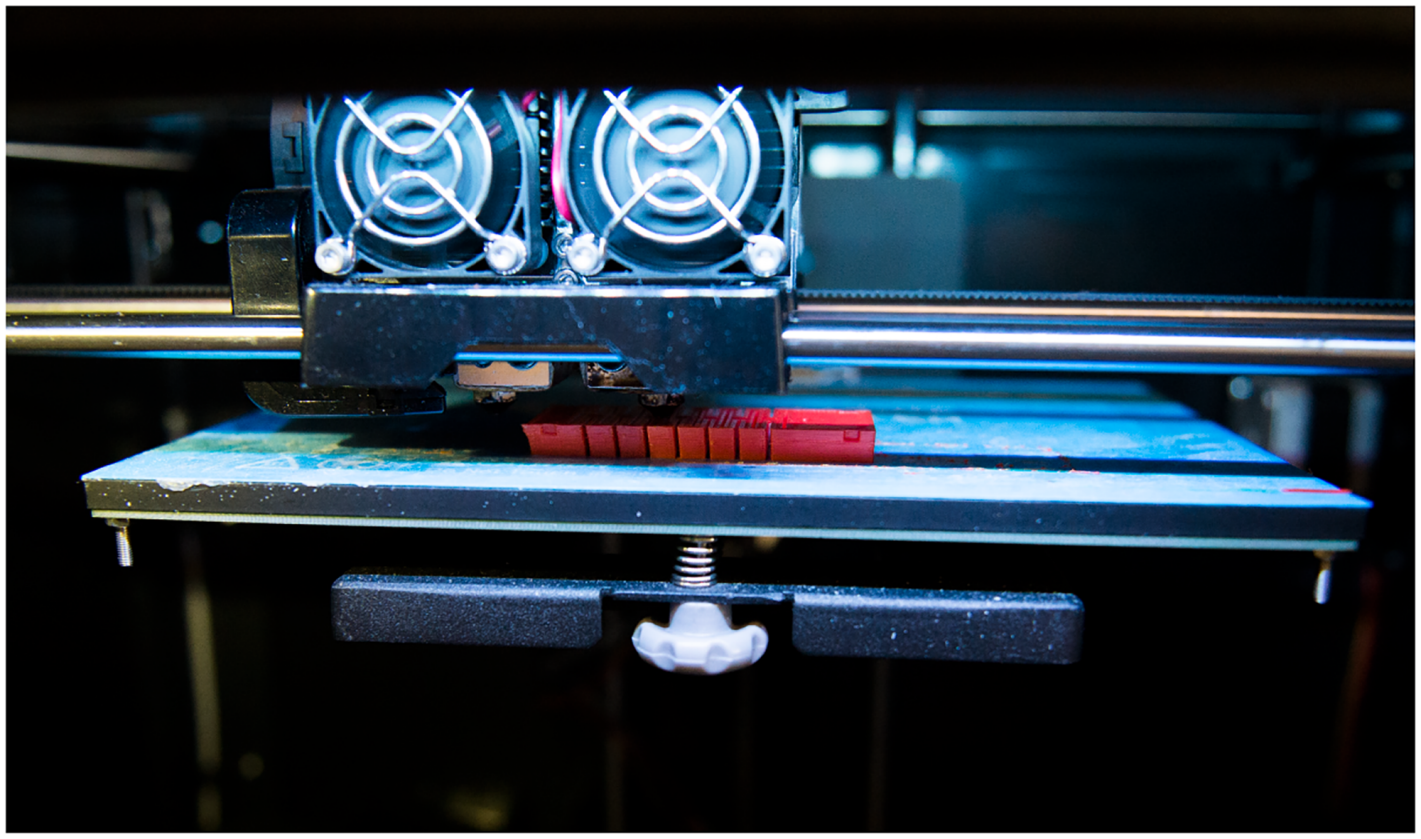
3D printing soft actuators. Example of printing a soft bellows out of TPU.

Two off-the-shelf 3D printers (FlashForge Creator Pro, City of Industry, CA) were used to allow modification and manufacture of new manipulators at sea. The materials used for printing were Thermoplastic Polyurethane (TPU Ninjaflex, Ninjatek, St. Manheim, PA, USA) for flexible parts and PLA (Hatchbox, Pomona, CA, USA) for rigid parts. Fig 6 shows an example of 3D printing a soft bellows mechanism out of flexible material. The parts were designed using Computer Aided Design (CAD) software (Fusion 360, Autodesk, Mill Valley, CA, USA) and converted to 3D models (“.stl”) files in machine code for the 3D printer using a slicing software (Simplify 3D, Cincinnati, OH, USA) which generated a machine code file (“.x3g”) for the 3D printer. The ship encountered moderate seas (4m on some days), but the ship’s pitch and roll did not impact the quality of the 3D printing.

#### Entirely 3D printed gripper

**Fig 7:**
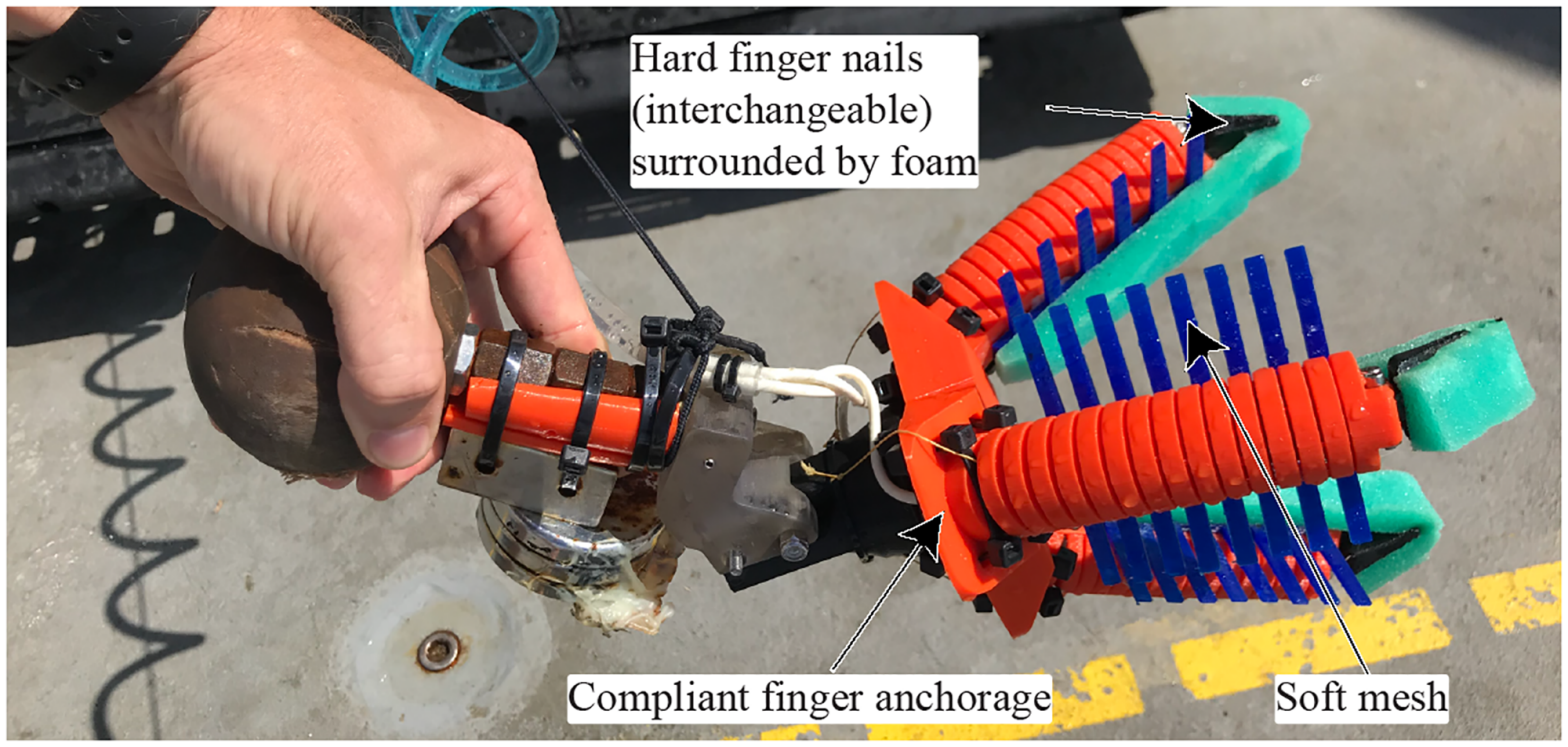
Fully 3D printed soft manipulator. The orange and blue parts are printed with (flexible) TPU, black parts are printed with (hard) PLA.

The 3D printed version shown in Fig 7 addresses several challenges that were revealed during the first dives. At first, the overall robustness of the soft manipulator was improved by using a compliant palm printed with the flexible material. The fingers mated with the palm and locked to position by press-fit and zip-ties. These changes allowed more forgiveness when colliding the manipulator with surrounding rocks or objects on the ROV’s forward tray.

Another at-sea modification was the addition of interchangeable “fingernails” to the soft gripping fingertips. These fingernails were printed out of both hard and soft material and allowed for better under-grasping when the specimen was located on hard substrata [24]. To protect the specimen during sampling, a layer of porous foam was added along the finger and around the nail. Finally, a flexible mesh was added on each finger to allow for additional contact points on the sample. Both the foam and the mesh were bonded using a flexible adhesive (Vinyl, Fabric & Plastic Flexible Adhesive, Loctite, Rocky Hill, CT, USA) which results in, after a full cure of 24h, a transparent and waterproof bond.

Because these manipulators are pressure-driven, it is critical to ensure there are no leaks after 3D printing. Key parameters in S1 Table demonstrate the layer height and extrusion temperature used during the print to mitigate any leakage. It is also important that 3D printed parts sent to depth are printed with a 100% infill ratio, to prevent implosion due to compression of trapped air inside the structure.

#### Multi-mode gripper

**Fig 8:**
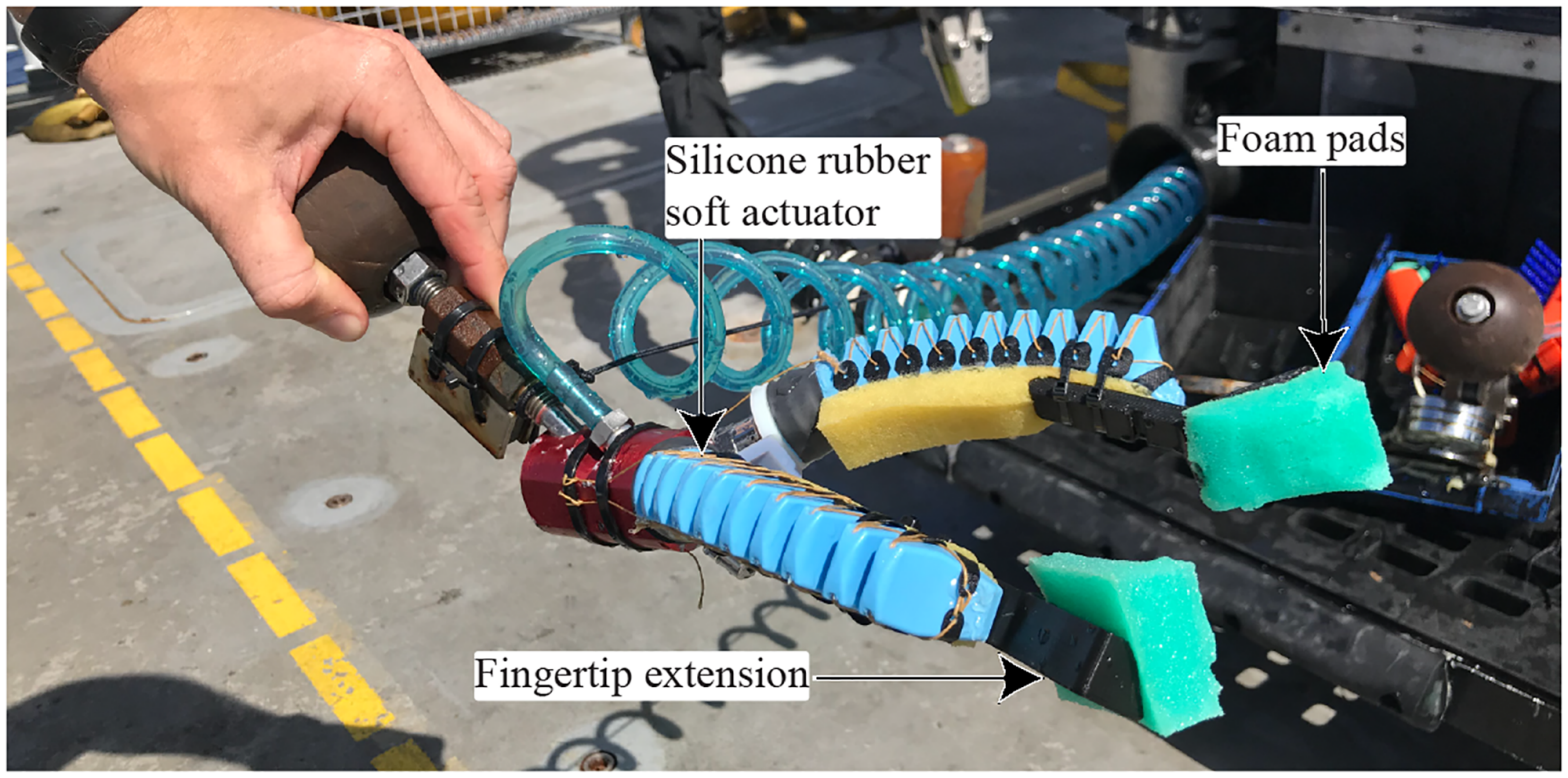
Modified three fingers soft manipulator. Modified three-finger soft manipulator converted to a two-finger version to allow pinch and power grasps.

Feedback from the ROV pilots and scientists also led to a modification of the finger arrangement in the gripper. After removing one of the fingers, a 3D printed fingertip extension was added with foam pads as shown in Fig 8.

**Fig 9:**
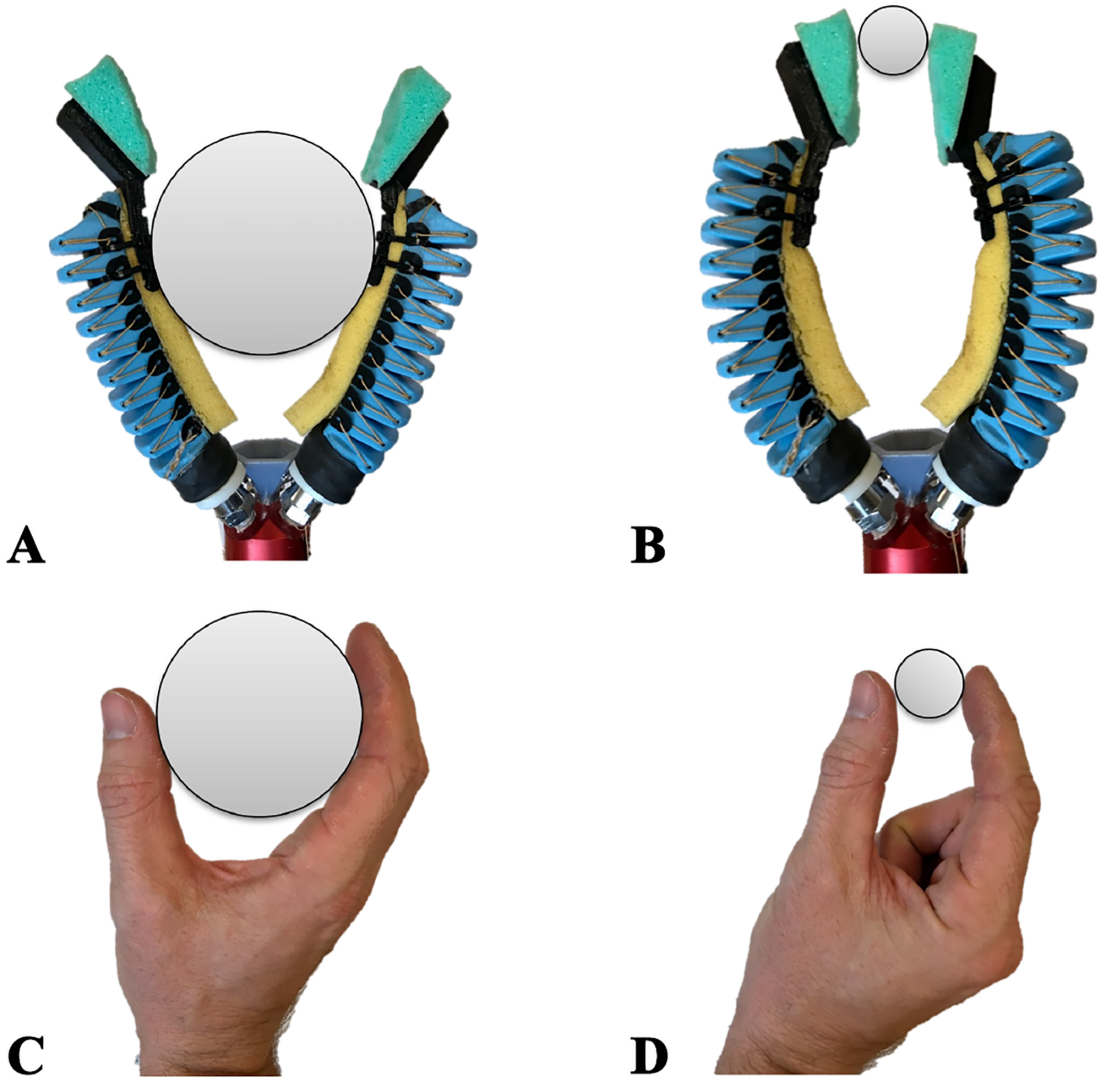
Various types of grasping and comparison with a human hand. A & C: A power grasp allows to pick up large objects. B & D: Pinch grasp allows more dexterity to pick up small objects.

This modification had several advantages. First, two-fingered soft grippers more closely resemble existing gripper designs typically installed on most ROVs, making them more intuitive to use for the ROV pilots. Secondly, the foam pads and finger orientation allowed for better pinching grasps. This gave more versatility to the manipulator, making it capable of both power grasps (Fig 9A and 9C) and pinch grasp (Fig 9B and 9D).

## Results and discussion

### First dives and in-field challenges

S2 Table summarizes the ROV dives executed during the expedition. The first few dives were used to evaluate the grasping potential and limitations of both the existing hard grippers and suction sampler on the ROV, as well as the initial version of the soft manipulators.

### Challenges with ROV tools

**Fig 10:**
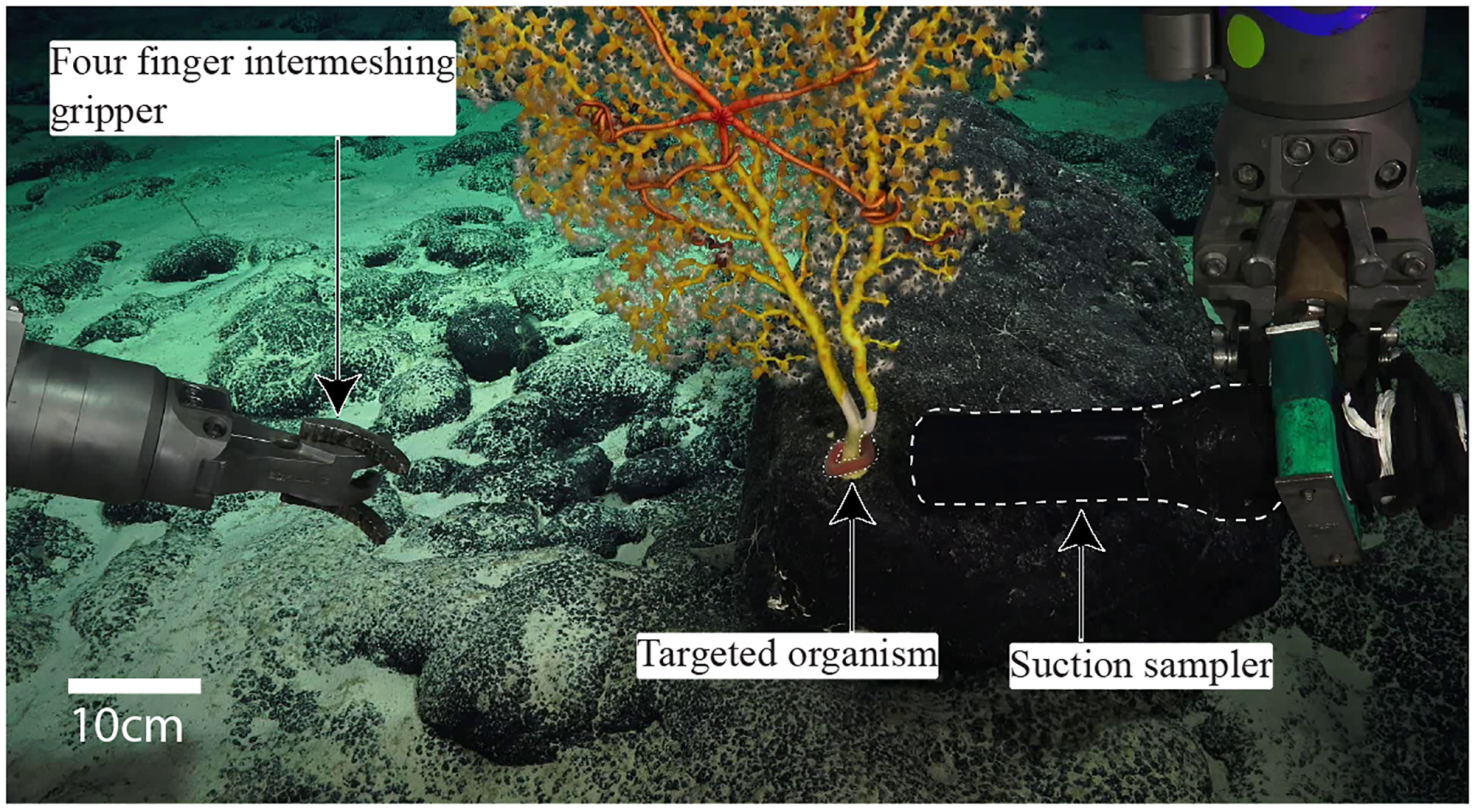
A challenging grasping situation. An aplacophoran mollusc at the base of a delicate coral was difficult to grasp without damaging the coral.

In several instances, the ROV’s standard toolkit (claws and suction sampler) was insufficient to collect desired organisms. For example, an aplacophoran mollusc wrapped around the base of a bamboo coral skeleton partially overgrown by a zoanthid (*Gerardia* sp.) soft coral (Fig 10) was not possible to collect without damaging the host coral. The flow generated by the suction sampler lacked the power needed to loosen the mollusc and attempts to obtain with the port gripper would have damaged the coral.

**Fig 11:**
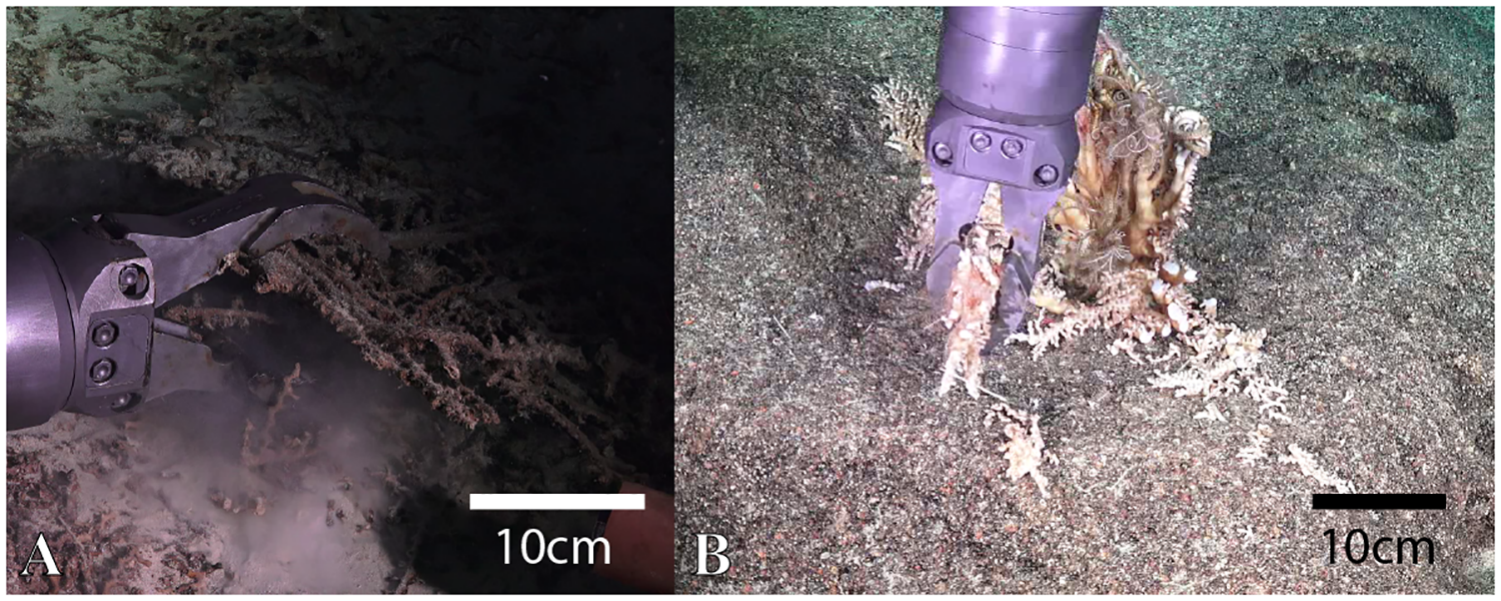
Challenges when grasping brittle specimens with hard bodied manipulators. A: Coral rubble (depth: 616m, S1 Video). B: *Enallopsammia* sp. coral (depth: 434m).

Picking up delicate samples is a challenge using the rigid manipulator, due to the lack of haptic feedback and the strong forces that the arm can generate. Additionally, non-stereoscopic vision makes the positioning and alignment of the arm difficult. Fig 11A shows an example of sampling coral rubble (S1 Video) and Fig 11B shows the sampling of the colonial scleractinian (hard) coral *Enallopsammia* sp. Although some samples were successfully collected with the rigid manipulator, they were often damaged during the process.

### Challenges with laboratory-fabricated soft manipulator

**Fig 12:**
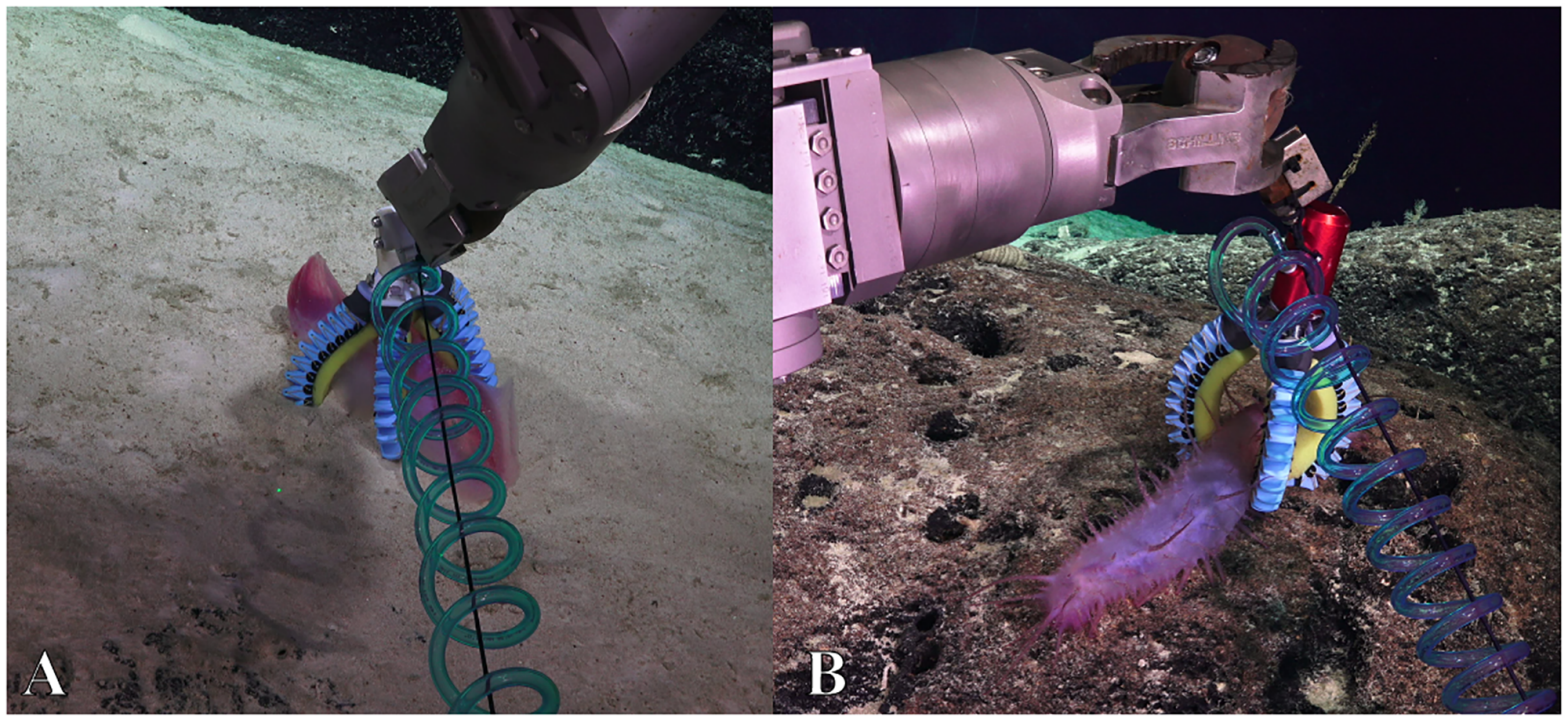
Examples of grasping sea cucumber (Holothuria). A: On a sandy substrate (depth: 2224m). B: On a rocky substrate (depth: 1282m).

The first prototypes of our soft manipulators confronted several challenges: a) some samples were difficult to grasp from underneath due to their location on hard, volcanic substrates and b) some organisms were often sessile-attached to rocky substrates compared to sandy bottoms (Fig 12A, S2 Video). For example, attempts to grasp a holothurian (Fig 12B, S3 Video), firmly adhered to a rock, were unsuccessful due to the rocky substrate and strong grip of the benthic organism.

**Fig 13:**
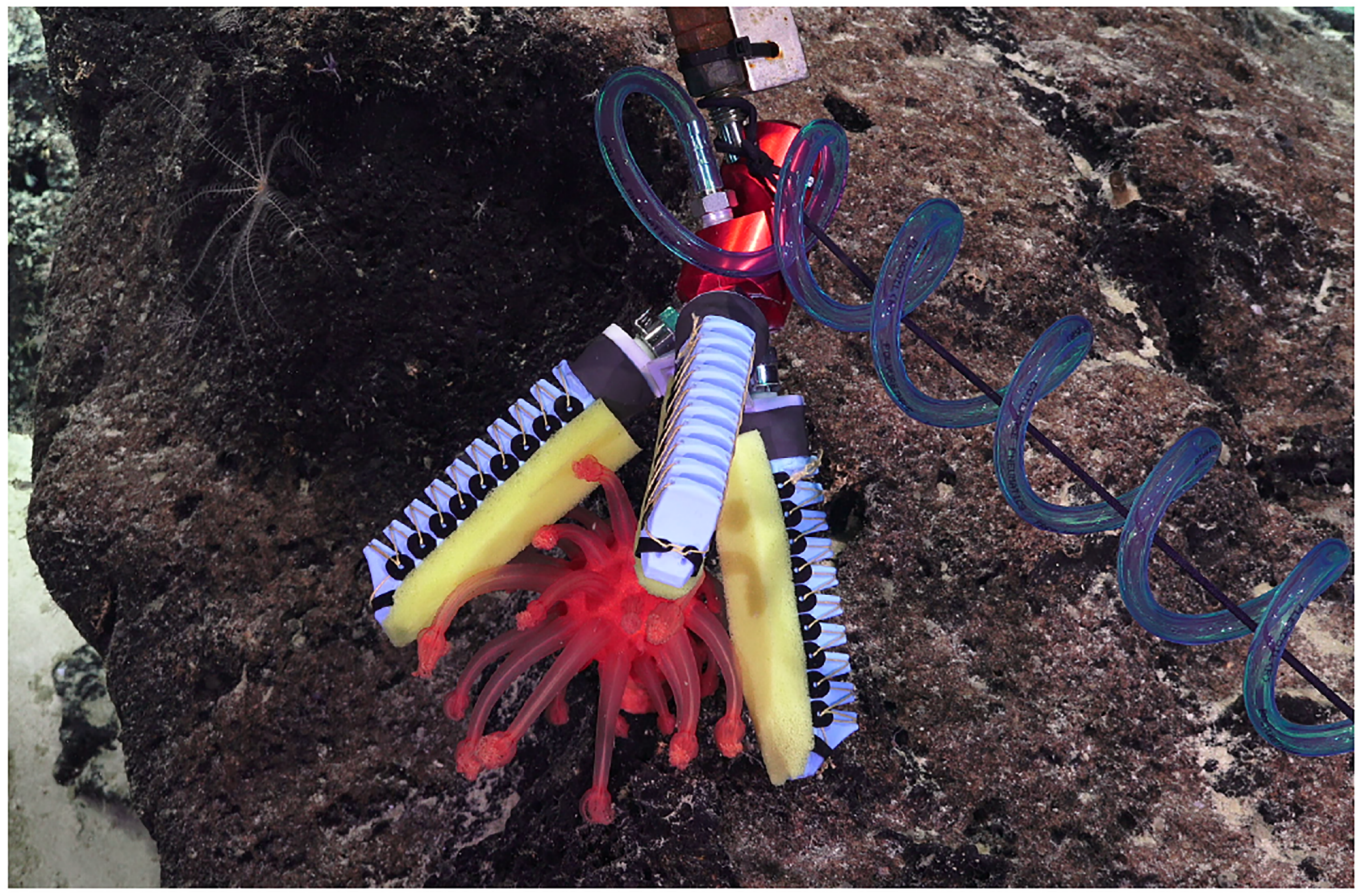
Difficulties in grasping. Example of orienting the manipulator horizontally or perpendicularly from a deep-sea mushroom coral (*Anthomastus* sp., depth: 1282m).

During the initial dives, training was also required for the ROV pilots to become familiar with the soft manipulators. ROV pilots tended to orient the manipulator horizontally or perpendicular to the sample as shown in Fig 13. This occurred due to the constraints of positioning the robot arm or due to the habit of using bilaterally symmetrical, hard grippers. Real-time communication between ROV pilots, engineers, and biologists was a necessary component of successful trials, as it allowed the ROV pilots to adapt to the new and evolving soft manipulators.

### Ad-hoc 3D printed grippers

#### Entirely 3D printed gripper

**Fig 14:**
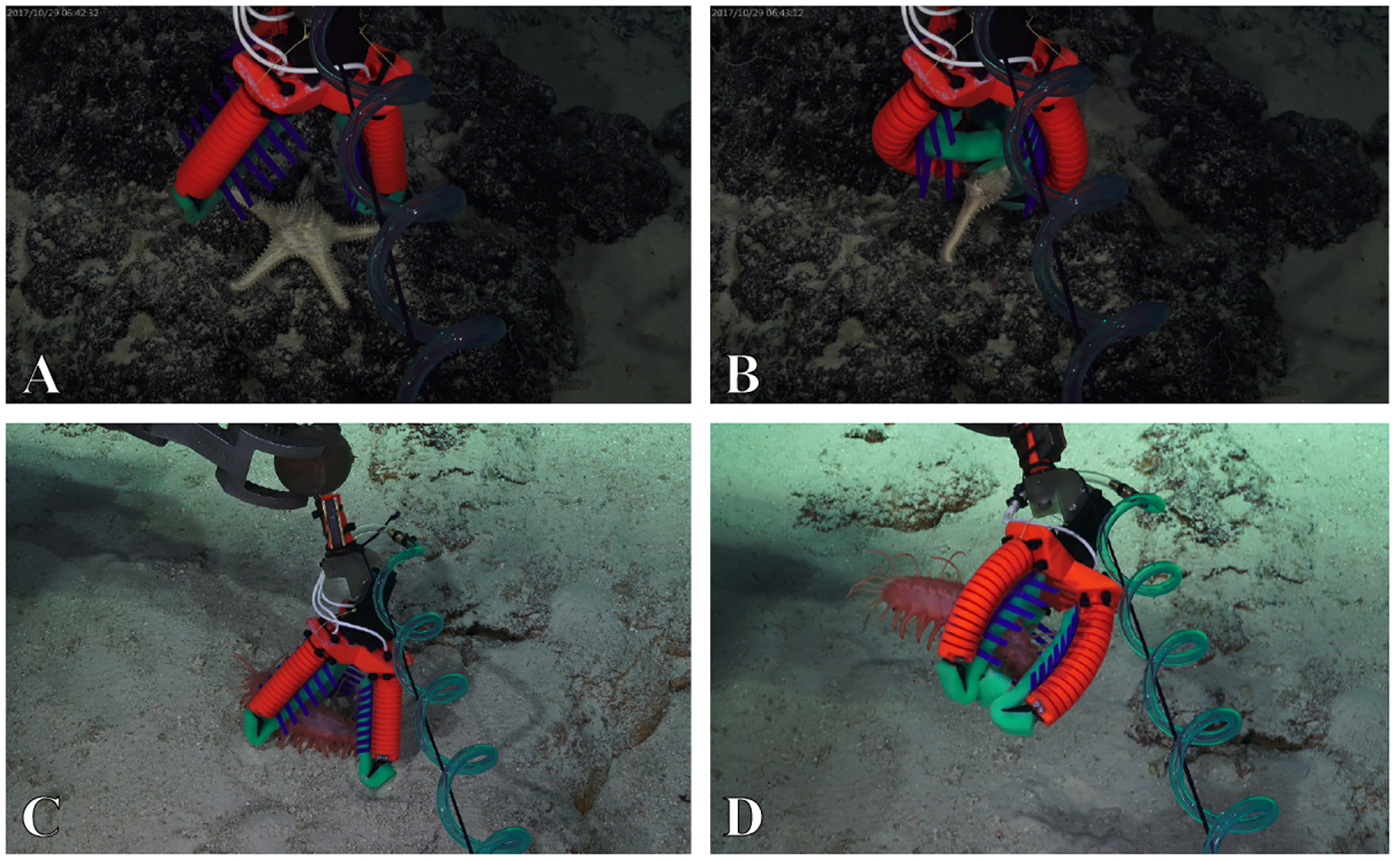
Sampling with a 3D printed soft manipulator designed and constructed on-board the ship. A & B: a goniasterid (depth: 1162m). C & D: a holothurian (depth: 843m).

During dive SB0083 (Carondelet Reef, at a maximum depth of 1473m), the newly designed 3D printed soft manipulator was utilized on two successful collections. At first, a goniasterid (Fig 14A and 14B, S4 Video) positioned on a rock was collected. This is a typical example of a sample that would be severely damaged if a hard-bodied manipulator was utilized. The second grasp was a holothurian (Fig 14A and 14B) lying on sand. It was picked up and released to demonstrate the benefits of the soft mesh in delicately holding the organism (S5 Video).

#### Multi-mode gripper

**Fig 15:**
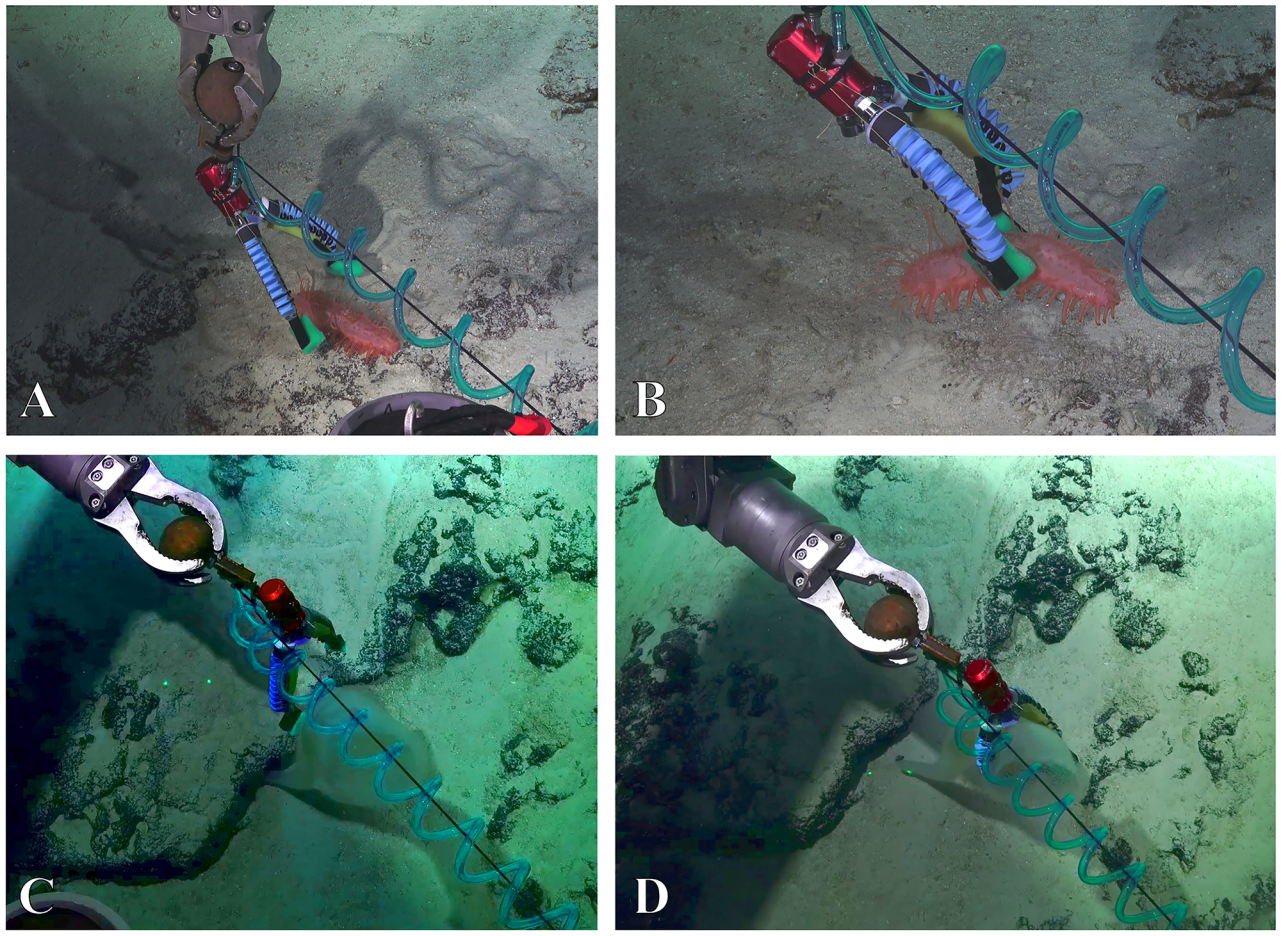
The multi-mode gripper could be used successfully for sampling several sea creatures. A & B: pinch grasp on a holothurian (depth: 843m). C & D: a power grasp on a hexactinellid sponge (depth: 1361m).

The two-fingered gripper was tested during the final dive. A pinch grasp was demonstrated with a sea cucumber (Fig 15A and 15B, S6 Video). The grip was firm despite shaking the end-effector. When engineering tests were complete, the sample was delicately replaced on the ground. During a second grasping opportunity, a power grasp was demonstrated on a hexactinellid glass sponge (Fig 15C and 15D, S7 Video). This grasp was extremely challenging because the ROV was hovering above the organism. Nevertheless, the organism was successfully and gently grasped and released by the manipulator.

With these new modifications, this multi-mode gripper would have been highly likely to successfully grasp the aplacophoran mollusc on the coral base (Fig 10) or to successfully grasp brittle coral rubble. Indeed, the two fingers were demonstrated to easily surround cylindrical objects.

## Conclusion and future directions

We have described the design, fabrication, on-the-fly modifications of, and improvements to soft robotic manipulators on an oceanographic research cruise to one of the most remote regions of the Pacific Ocean. This tool kit offers purpose-built modes for gently interacting with fragile marine life. The standard hard robotic claws and slurping or suction technologies are applicable to a subset of organisms, but more delicate life forms are often excluded from deep sea biological diversity surveys as the tools necessary to interact with them are not feasible. Soft robotics provides the advantage of delicate interactions and manipulations to enable previously-impossible sampling strategies. By 3D-printing soft robotics on-board the research vessel during an active scientific expedition, we have shown that we can increase our ability to study biodiversity in a remote and resource-limited environment. Each soft-robotic device is also applicable to a range of sizes, morphologies and motilities. This *ad-hoc* soft robotic printing of custom manipulators reduces the time and money necessary to revisit remote research sites, as scientists do not need to return to a land-based research laboratory to engineer new sampling tools. Our successful tests of soft robotics sets the foundation for the development of embedded sensors and lensless imaging technology to investigate physiological parameters *in situ* and provide 3D reconstructions of deep-sea organisms, while leaving them unharmed.

## Supporting information

S1 Table 3D printing parameters for flexible and hard materials. (PDF)

S2 Table Summary of all the dives. (PDF)

S1 Video Challenges when grasping brittle specimens with hard bodied manipulator. Example with coral rubble (depth: 616m).
(MP4)

S2 Video Example of grasping a sea cucumber. On a sandy substrate (depth: 2224m).
(MP4)

S3 Video Example of grasping a sea cucumber. On a rocky substrate (depth: 2224m).
(MP4)

S4 Video Sampling with a 3D printed soft manipulator designed and constructed on-board the ship. Sampling a goniasterid (depth: 1162m).
(MP4)

S5 Video Sampling with a 3D printed soft manipulator designed and constructed on-board the ship. Sampling a holothurian.
(MP4)

S6 Video Multi-mode gripper. Pinch grasp on a holothurian.
(MP4)

S7 Video Multi-mode gripper. Power grasp on a hexactinellid sponge.
(MP4)

S1 Fig Soft actuator characterization. All soft manipulators were tested on a material tester (Instron 5544A, Instron, Norwood, MA 02062, USA). The actuators were fixed at their base, and oriented downwards on a load cell. Pneumatic pressure was applied up to 140kPa (a typical pressure used when grasping an object) to the actuator and the blocked force was recorded. For each actuator type, the experiment was repeated three times; markers indicate the mean values (circles) and standard deviations (shaded area). The images (right) show the actuators under the minimum (0kPa) and maximum (140kPa) pressure: (a) 3D printed with fingernails, (b) 3D printed without fingernails, (c) lab molded without extension, and (d) lab molded with extension.
(TIF)
