# Supplementary material for: Shipboard design and fabrication of custom 3D-printed soft robotic manipulators for the investigation of delicate deep-sea organisms
Source: PLoS One. 2018 Aug 1;13(8):e0200386. doi: 10.1371/journal.pone.0200386 (PMC6070194; doi:10.1371/journal.pone.0200386)
Supplement: S1 Table — (PDF) [file pone.0200386.s001.pdf]

S2 Table: 3D printing parameters

Parameter	Value for soft material (TPU)	Value for hard material
Layer height	0.1mm	0.2mm
Extrusion Temperature	220C	220C
Bed Temperature	80C	80C
Print speed	20mm/s	80mm/s
Infill	100%	100%
Wall thickness	2mm	-
Cooling	60%	60%
Multiplier number	1.2	1.0
Support	None	None
Retraction	None	2mm
