# Supplementary material for: Shipboard design and fabrication of custom 3D-printed soft robotic manipulators for the investigation of delicate deep-sea organisms
Source: PLoS One. 2018 Aug 1;13(8):e0200386. doi: 10.1371/journal.pone.0200386 (PMC6070194; doi:10.1371/journal.pone.0200386)
Supplement: S2 Table — (PDF) [file pone.0200386.s002.pdf]

S1 Table: Summary of all the dives

Dive ID	Max Depth	Gripper 1	Gripper 2	Sample	Grasping purpose	Grasp Successs
SB0067	N/A	3 fingers	5 fingers	N/A	N/A	N/A
SB0068	2021m	3 fingers	5 fingers	N/A	N/A	N/A
SB0069	2294m	3 fingers	5 fingers	N/A	N/A	N/A
SB0070	1189m	3 fingers	5 fingers	N/A	N/A	N/A
SB0071	1725m	3 fingers	5 fingers	N/A	N/A	N/A
SB0072	1430m	5 fingers	N/A	Deep sea Anemone	Catch-and-release	No
		5 fingers	N/A	Sea cucumber (Holothuria)	Catch-and-release	No
SB0073	2440m	5 fingers	N/A	Sea cucumber (Holothuria)	Catch-and-release	Yes
		5 fingers	N/A	Deep sea Anemone	Catch-and-release	No
SB0074	1361m	Experimental gripper #1	N/A	N/A	N/A	N/A
SB0075	643m	4 fingers	N/A	Coral rubble	Post-study	Yes
		4 fingers	N/A	Coral	Post-study	No
SB0076	1517m	N/A	N/A	N/A	N/A	N/A
SB0077	1424m	3 fingers	none	Coral rubble	Post-study	Yes
SB0078	1378m	Experimental gripper #1	**3 fingers**	Hard coral	Post-study	No
		Experimental gripper #1	**3 fingers**	Sponge	Post-study	Yes
		**Experimental gripper #1**	3 fingers	Crinoid	Catch-and-release	No
		**Experimental gripper #1**	3 fingers	Sponge	Catch-and-release	No
SB0079	1958m	**3 fingers**	Experimental gripper #2	Acorn worm	Post-study	No
		3 fingers	**Experimental gripper #2**	Coral	Post-study	No
SB0080	829m	**3 fingers**	Experimental gripper #2	Fossil shelves	Post-study	Yes
SB0081	1028m	**3 fingers**	Experimental gripper #1	Crinoid	Catch-and-release	No
		**3 fingers**	Experimental gripper #1	Anemone	Catch-and-release	Partial
		**3 fingers**	Experimental gripper #1	Shrimp	Catch-and-release	No
		**3 fingers**	Experimental gripper #1	Rock	Post-study	Partial
SB0082	241m	-	-	-	-	-
SB0083	1473m	**3D printed manipulator**	2 fingers adapted	Sea star (Asteroidea)	Post-study	Yes
		**3D printed manipulator**	2 fingers adapted	Sea cucumber (Holothuria)	Catch-and-release	Yes
		3D printed manipulator	**2 fingers adapted**	Sea cucumber (Holothuria)	Catch-and-release	Yes
		3D printed manipulator	**2 fingers adapted**	Hexactinellid sponge	Catch-and-release	Yes

- ”# fingers” grippers denotes silicone based actuators fabricated in laboratory.
- The manipulator in use for a sampling is indicated in bold.
- A partial grasp success indicates that the sample was successfully grasped but fell during the transport to the biobox/quiver. In most of the cases it could be recovered later on.
- A post-study grasping purpose indicates that the sample is intended to be taken back to the surface for further biological study.
