# Supplementary figures and images for: Shipboard design and fabrication of custom 3D-printed soft robotic manipulators for the investigation of delicate deep-sea organisms
Source: PLoS One. 2018 Aug 1;13(8):e0200386. doi: 10.1371/journal.pone.0200386 (PMC6070194; doi:10.1371/journal.pone.0200386)

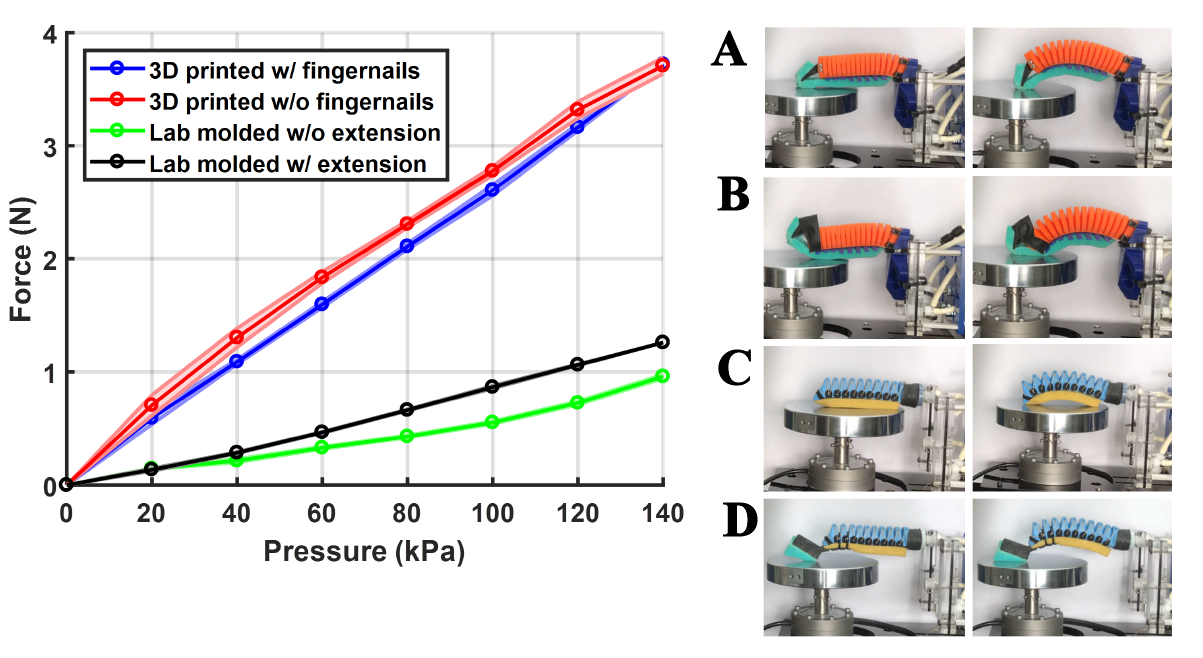

Supplement: S1 Fig — All soft manipulators were tested on a material tester (Instron 5544A, Instron, Norwood, MA 02062, USA). The actuators were fixed at their base, and oriented downwards on a load cell. Pneumatic pressure was applied up to 140kPa (a typical pressure used when grasping an object) to the actuator and the blocked force was recorded. For each actuator type, the experiment was repeated three times; markers indicate the mean values (circles) and standard deviations (shaded area). The images (right) show the actuators under the minimum (0kPa) and maximum (140kPa) pressure: (a) 3D printed with fingernails, (b) 3D printed without fingernails, (c) lab molded without extension, and (d) lab molded with extension. (TIF) [file pone.0200386.s010.tif]
